# Evaluating Sleep Interventions in Psychosis and Schizophrenia: A Systematic Review of the Efficacy and Effectiveness

**DOI:** 10.1192/bjo.2026.11103

**Published:** 2026-06-30

**Authors:** Sajitha Nair, Kazi Shams, Harkeerat Maan, Adekemi Oluwabunmi, Sukhi Shergill

**Affiliations:** 1 Kent and Medway Mental Health Trust, Ashford, United Kingdom; 2 Kent and Medway Mental Health Trust, Maidstone, United Kingdom; 3 Kent and Medway Mental Health Trust, Canterbury, United Kingdom

## Abstract

**Aims:**

To evaluate the impact of sleep interventions on sleep quality, duration, and efficiency in adults with psychotic disorders, and to assess secondary effects on psychotic symptoms, daytime functioning, and mental health stability.

**Methods:**

A systematic review of studies published in the past 10 years was conducted, including meta-analysis, randomized and non-randomized trials, and observational studies. Eligible studies involved adults (*≥*18 years) with psychotic disorders and documented sleep disturbances. Interventions included pharmacological agents (melatonin, Z-drugs, antipsychotics), psychological therapies (CBT-I), and other complementary therapies (light, relaxation).

**Results:**

Twenty-three studies met inclusion criteria. Cognitive Behavioural Therapy for Insomnia (CBT-I), was the most frequently studied and consistently effective intervention across delivery formats. Pharmacological agents offered variable short-term benefits but raised concerns about side-effects, tolerance, and dependency. Adjunctive therapies such as music therapy, relaxation and exercise, light-dark rhythm modulation etc. demonstrated promise in specific subpopulations. Common limitations included methodological heterogeneity, small samples, and limited long-term follow-up.

**Conclusion:**

CBT-I, adapted for psychotic disorders, appears the most robustly supported intervention. Integrated approaches combining psychological, pharmacological, and adjunct therapies may optimise outcomes. Large-scale, long-term studies and pathways for routine care integration are warranted, with inclusion in national guidelines.

